# Super-barcoding of four *Agrimonia* species distributed in Korea based on complete plastid genomes and nuclear ribosomal DNAs

**DOI:** 10.1371/journal.pone.0341151

**Published:** 2026-02-13

**Authors:** Jae-Joon Lee, Jong-Soo Kang, Yeon Jeong Kim, Yun Sun Lee, Jee Young Park, Woojong Jang, Byeong Cheol Moon, Yongduk Kim, Tae-Young Kim, Tae-Jin Yang

**Affiliations:** 1 Department of Agriculture, Forestry and Bioresources, Plant Genomics & Breeding Institute, Research Institute of Agriculture and Life Science, College of Agriculture & Life Sciences, Seoul National University, Seoul, Republic of Korea; 2 Department of Forest Resources, College of Forest and Environmental Sciences, Kangwon National University, Chuncheon, Republic of Korea; 3 BK21 Agriculture-forestry Bioresource Convergence Center, Seoul National University, Seoul, Republic of Korea; 4 Crop Biotechnology Institute, Institutes of Green-bio Science and Technology, Seoul National University, Pyeongchang, Republic of Korea; 5 Herbal Medicine Resources Research Center, Korea Institute of Oriental Medicine, Naju, Republic of Korea; 6 BTC Corporation, Ansan, Gyeonggi, Republic of Korea; Institute for Biological Research, University of Belgrade, SERBIA

## Abstract

The genus *Agrimonia* is widely distributed throughout temperate regions and includes species used in traditional medicine in Asia and Europe. However, their accurate identification is often challenging because the vegetative parts used, such as leaves and roots, are morphologically highly similar across species. To investigate the genetic diversity of *Agrimonia* species commonly distributed and traded in Korea and to develop reliable molecular tools for species authentication, we collected 36 samples primarily representing four *Agrimonia* species (*A. pilosa, A. coreana*, *A. nipponica*, and *A. eupatoria*). We sequenced and assembled complete plastid genomes (plastomes) and 45S nuclear ribosomal DNA (nrDNA) sequences from these four species. The assembled plastomes ranged from 155,128–155,313 bp, while the nrDNA sequences spanned 5,860–5,873 bp. Phylogenetic analyses based on both plastome and nrDNA datasets revealed that each species formed a distinct clade, demonstrating clear genetic differentiation among taxa. Based on plastome sequence variations, we developed eight plastome-based super-barcoding markers and validated their reliability using 36 *Agrimonia* accessions, including an additional closely related congeneric accession, *A. gorovoii*. The markers successfully classified samples into species-specific haplotype groups. This plastome-based super-barcoding approach provides a practical molecular authentication method for major *Agrimonia* species used as medicinal resources in Korea, thereby facilitating quality control and accurate utilization of *Agrimonia* materials.

## Introduction

The genus *Agrimonia*, belonging to the family Rosaceae, includes *A. pilosa*, a species long used in traditional medicine in East Asia for treating diarrhea and bleeding [1]. Recent studies have highlighted its potential antioxidant, anti-inflammatory, and anti-tumor properties [1–3]. Beyond medicinal use, *Agrimonia* species are also utilized in cosmetic formulations, such as skin whitening and anti-wrinkle effects, owing to their rich polyphenolic content [3]. In Korea and China, only certain *Agrimonia* species such as *A. pilosa* and *A. eupatoria* are listed in national pharmacopoeias as approved medicinal materials. Because only a limited number of *Agrimonia* species are pharmacologically recognized and officially regulated, strict quality control and accurate species authentication are essential to ensure the safety and efficacy of *Agrimonia*-based medicinal products. In particular, four species, *A. pilosa*, *A. coreana*, *A. nipponica*, and *A. gorovoii*, are native to Korea [4,5], where they are traditionally utilized and frequently appear in the herbal trade. Although *A. eupatoria* does not naturally occur in Korea, it is widely used as a medicinal substitute and is commercially imported for pharmaceutical and herbal applications.

Despite its medicinal importance, the identification of *A. pilosa* remains challenging. Although this species is characterized as an octoploid (2n = 8x = 56) with 10–17 stamens [1], it shares highly similar vegetative characteristics with closely related species such as *A. coreana*, *A. nipponica*, *A. eupatoria*, and *A. gorovoii* [4,5]. Species identification within this group relies largely on reproductive traits, particularly the number and morphology of stamens and floral structures, rather than vegetative morphology [4,5]. Consequently, accurate identification becomes extremely difficult or even impossible in the absence of reproductive organs, especially during non-flowering seasons. Moreover, morphologically similar species such as *A. coreana* and *A. nipponica* are frequently misidentified or substituted for *A. pilosa* in herbal trade, which may compromise product efficacy and safety. This problem is further complicated by the fact that these plants are often traded in commercial markets as dried leaves, stems, or roots, making morphological identification nearly impossible. Compounding these issues, limited genetic information is available for the genus *Agrimonia*, which necessitates the collection and genetic analysis of a comprehensive set of accessions. With such comprehensive genetic information, the development of reliable molecular markers represents a more straightforward and reliable approach to species authentication [6–8].

Under these circumstances, an effective alternative for species identification is molecular discrimination using DNA markers. In plants, molecular identification has traditionally relied on a small number of plastid markers such as *matK, rbcL,* and *trnH-psbA* [9,10]. However, a limited number of plastid regions frequently lack sufficient resolution to discriminate among closely related species [11]. As an alternative, the super-barcoding approach that utilizes the complete plastome has recently gained attention for its high resolution in detecting interspecific variation [12]. The *de novo* assembly using Low-Coverage Whole-genome sequencing (dnaLCW) method enables cost-effective plastome assembly from only 1–2 Gbp of next-generation sequencing (NGS) data [13]. This method also allows for the concurrent analysis of the 45S nuclear ribosomal DNA (nrDNA) and provides a cross-validation of data via read-mapping depth. As the number of plastome sequences in public databases such as GenBank increases, the utility and applicability of such methods continue to expand.

This super-barcoding approach enables more accurate species identification compared to a small set of conventional plastid markers. Given the taxonomic complexity and frequent misidentification within the genus, this study aims to establish a practical molecular authentication system for *Agrimonia* species commonly used and traded in Korea, where species misidentification frequently occurs due to morphological similarity. Therefore, to clarify species boundaries among *Agrimonia* species distributed in Korea, we assembled complete plastome and 45S nrDNA sequences from four representative species (*A. pilosa, A. coreana, A. nipponica* and *A. eupatoria*). Based on comparative plastome analyses, we designed species-discriminatory plastome-based markers and evaluated their applicability using a total of 36 *Agrimonia* accessions collected from Korea, including an additional closely related congeneric accession, *A. gorovoii*. Such approaches are particularly effective when applied to regionally defined taxonomic problems involving closely related medicinal plant species.

## Materials and methods

### Plant collection

Four wild-collected samples of *Agrimonia* (two from *A. pilosa*, one from *A. coreana,* and one from *A. nipponica*), representing three species, were obtained from different locations in South Korea (Table 1, S1A-D Fig in S2 File). One *A. eupatoria* sample was purchased from a commercial supplier in Bulgaria. These five samples were authenticated at the time of collection based on morphological characteristics, including flower size and stamen number, and were subsequently used for plastome and nrDNA analysis. No permission was required for the collection of these wild samples in accordance with the respective national and local legislations. An additional 31 *Agrimonia* samples used for DNA marker validation were collected from wild populations and provided by BTC Corporation (Ansan, South Korea), the National Institute of Biological Resources (NIBR, Incheon, South Korea), and the Korea Institute of Oriental Medicine (KIOM, Naju, South Korea) (S3 Table in S1 File). Although plastome and 45S nrDNA sequences of *A. gorovoii* could not obtained due to DNA degradation in the available material, this species was intentionally included in the marker validation step as an additional congeneric taxon to preliminarily assess the specificity of the plastome-based markers developed in this study (S1E Fig in S2 File).

**Table 1:** Summary of sample information and assembly statistics for the five *Agrimonia* plastomes and 45S nrDNAs.

Species (Sample name)	Collection site	Accesion number (cp/nrDNA)	Raw data (Gbp)	Plastome		45S nrDNA	
**Coverage (**×)	**Length (bp)**	**Coverage (**×)	**Length (bp)**
*Agrimonia pilosa* (*A. pilosa*-1)	Chilgok, South Korea	PQ634271 / PQ249110	1.49	57.27	155,144	434.72	5,860
*Agrimonia pilosa* (*A. pilosa*-2)	Hongcheon, South Korea	PQ634272 / PQ249111	1.59	37.51	155,128	506.43	5,860
*Agrimonia coreana*	Gapyeong, South Korea	PQ634273 / PQ249112	1.88	97.02	155,313	744.49	5,873
*Agrimonia nipponica*	Geoje, South Korea	PQ634274 / PQ249113	1.42	64.47	155,162	922.54	5,863
*Agrimonia eupatoria*	Bulgaria, Europe	PQ634275 / PQ249114	1.86	168.72	155,265	372.26	5,861

### DNA Extraction, sequencing, and annotation of plastome and 45S nrDNA

Total genomic DNA was extracted from cauline leaves using the GeneAll^Ⓡ^ Exgene^TM^ Plant SV Mini Kit (GeneAll Biotechnology Ltd., South Korea) according to the manufacturer’s instructions. The quality and concentration of the DNA were evaluated using a NanoDrop ND-1000 (Thermo Fisher Scientific, USA). The extracted DNA was used for paired-end sequencing on the Illumina MiSeq platform (Illumina, Inc., USA) at Phyzen Co., Ltd. (South Korea). Raw reads were trimmed using the CLC quality trim (v4.06 beta.67189, CLC Inc., Denmark) with a Phred score threshold of ≥ 20.

After trimming, approximately 1–2 Gbp of high-quality reads per sample were used for the *de novo* assembly of plastome and 45S nrDNA sequences. Assembly was performed by the dnaLCW method [13] and the CLC genome assembler program (v4.6 beta, CLC Inc., Denmark) [14]. Assembly parameters, including overlapping distance, were explored within the range of 150–500 bp. Putative plastome contigs from the *de novo* assembly were identified and ordered by alignment to the previously reported *A. pilosa* plastome (GenBank accession No. NC_050051) [15] using MUMmer (v4.0.0. beta5, https://mummer4.github.io/). The ordered plastome contigs were joined to form a single draft sequence and then manually curated by mapping the trimmed short reads back to the draft sequence to resolve ambiguities. Similarly, the nrDNA contigs were generated using the 45S nrDNA sequence of *Spiraea prunifolia* (GenBank accession No. OP874593) as a reference [16].

The complete plastome sequences were annotated using GeSeq (https://chlorobox.mpimp-golm.mpg.de/geseq.html) [17], and annotations were manually corrected using Artemis (v3) [18]. A circular map of the plastomes was generated using OGDRAW (v1.3.1, https://chlorobox.mpimpgolm.mpg.de/OGDraw.html) [19]. The assembled 45S nrDNA sequences, including the 18S rRNA, 5.8S rRNA, 26S rRNA, and two internal transcribed spacer (ITS) regions, was annotated using BLAST (https://blast.ncbi.nlm.nih.gov/Blast.cgi). The complete plastome and 45S nrDNA sequences are deposited in NCBI GenBank, with accession numbers listed in Table 1.

### Phylogenetic tree construction

For the plastome analysis, 17 additional, previously assembled plastome sequences were downloaded from NCBI GenBank (S2 Table in S1 File). These included sequences from closely related species (used as outgroups) and other *Agrimonia* species. For the 45S nrDNA analysis, raw sequencing reads for four additional 45S nrDNA (comprising one *Robus hirsutus* as an outgroup and three *Agrimonia* species) were downloaded from Genbank (S2 Table in S1 File) and subsequently assembled using the methods described above. A total of 17 publicly available plastome sequences and four reassembled nrDNA sequences were combined with the five newly generated plastome sequences and five nrDNA sequences produced in this study. The resulting plastome dataset and the nrDNA dataset were then separately aligned using MAFFT (v7), with default parameter [20]. Phylogenetic trees for both alignments were then constructed using RAxML [21] with 1000 bootstrap replicates and the GTRGAMMA model of nucleotide substitution.

### Development of DNA markers

To discriminate among the four *Agrimonia* species analyzed at the plastome level (*A. pilosa*, *A. coreana*, *A. nipponica*, and *A. eupatoria*). An additional congeneric species was included in the validation step to preliminarily assess the specificity of the developed markers beyond the core target species. Plastome and nrDNA sequences of four *Agrimonia* species were aligned using MAFFT (v7) [20]. The resulting alignment was searched for interspecies or intraspecies polymorphisms, including single-nucleotide polymorphisms (SNPs) and insertions/deletions (InDels). InDel regions longer than 10 bp in the plastome alignment and three SNP regions were chosen as marker candidates. Primers for co-dominant InDel markers, dominant SNP markers, and co-dominant cleaved amplified polymorphic sequences (CAPs) markers were designed using Primer–BLAST [22] with default parameters. PCR amplification was conducted in 25 μL reactions using Taq DNA polymerase (Inclone Biotech, South Korea) according to the manufacturer’s instruction. The thermal cycling conditions were as follows: 94˚C for 5 min; 35 cycles of 94˚C for 20 sec, 56–58˚C for 20 sec, 72˚C for 30 sec; 72˚C for 5 min. Successful PCR amplification was verified by electrophoresis using a 3% (w/v) agarose gel.

## Results and discussion

### Assembly of plastome and 45S nrDNA sequences

**Fig 1:**
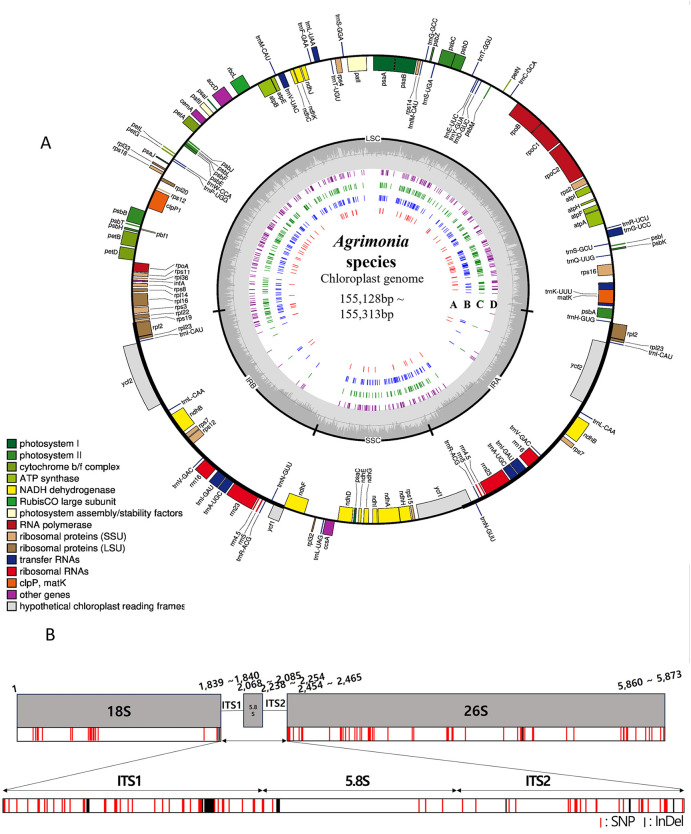
Genomic structure and sequence variation in the five assembled *Agrimonia* plastomes and 45S nrDNAs. **(A)** Circular gene map of the *Agrimonia* plastome. The inner variation tracks (labeled A–D) represent single nucleotide polymorphisms (SNPs) and insertions/deletions (InDels) in each sample relative to the one individual of *A. pilosa* (referred to as *A. pilosa*-1) reference sequence. Track A: *A. pilosa*-2; Track B: *A. coreana*; Track C: *A. nipponica*; Track D: *A. eupatoria*. Genes are shown as colored boxes, with colors correspond to their functional group. **(B)** Schematic diagram of the 45S nrDNA unit. Variable sites among the five samples were highlighted. Gray boxes indicate the transcribed regions, and red bars represent variable sites among the five samples. A close-up view at the bottom highlights the ITS1, 5.8S, and ITS2 regions, where most of SNPs and InDels is concentrated.

We sequenced the genomes of four *Agrimonia* species, including two individuals of *A. pilosa* (hereafter referred to as *A. pilosa*-1 and *A. pilosa*-2, respectively), and one individual each of *A. coreana*, *A. nipponica*, and *A. eupatoria* (S1 Table in S1 File). We then assembled their complete plastomes and 45S nrDNAs (Table 1). The resulting plastomes had varied lengths ranging from 155,128–155,313 bp and GC contents of approximately 36.9%, consistent with previous reports (Fig 1; Table 1 and S1 Table in S1 File) [23]. All plastomes showed a typical quadripartite structure, consisting of a large single copy (LSC) region of 84,439–84,959 bp, a pair of inverted repeats (IR) regions of 25,951–25,965 bp each and a small single-copy (SSC) region of 18,723–18,806 bp. Intraspecific variation was observed between *A. pilosa*-1 (155,144 bp) and *A. pilosa*-2 (155,128 bp). No differences in overall gene content or plastome structure were found among the five samples, which is consistent previous studies [23]. All the five *Agrimonia* plastomes contained 84 protein-coding genes, 37 tRNA genes, and eight rRNA genes (S1 Table in S1 File). The IR regions contained 18 duplicated genes, including seven protein-coding genes (*rpl2, rpl23, ycf2, ndhB, rps7, rps12,* and *ycf1*), four rRNAs (*rrn16, rrn23, rrn4.5,* and *rrn5*), and seven tRNAs (*trnI-CAU, trnL-CAA, trnV-GAC, trnI-GAU, trnA-UGC, trnR-ACG,* and *trnN-GUU*).

The total length of the assembled 45S nrDNA sequences ranged from 5,860–5,873 bp, with coverage of 372.26× to 922.54× (Fig 1B; Table 1). These sequences comprised the 18S, 5.8S, and 26S rRNA genes along with internal transcribed spacers (ITS1 and ITS2), forming the canonical structure of 45S nrDNA.

### Genetic diversity of plastomes and nrDNAs in the four *Agrimonia* species

To investigate genetic variations within and among species, we analyzed a total 16 plastomes. This dataset comprised the five newly assembled plastomes from this study and 11 publicly available sequences retrieved from GenBank (S2 Table in S1 File). The combined dataset included eight *A. pilosa,* two *A. coreana,* three *A. nipponica,* and three *A. eupatoria* accessions*.* One of the two *A. eupatoria* accessions was newly assembled in this study.

Pairwise comparisons of eight plastomes within *A. pilosa* revealed 14–64 SNPs and 1–40 InDels, with *A. pilosa*-1 and *A. pilosa*-2 differing by 64 SNPs and 31 InDels (Table 2). These results suggest a relatively low level of genetic diversity among Korean *A. pilosa* accessions, compared to the broader variation observed across geographic ranges in previous studies.

**Table 2:** Pairwise comparison of variable sites (SNPs and InDels) among *Agrimonia* plastomes.

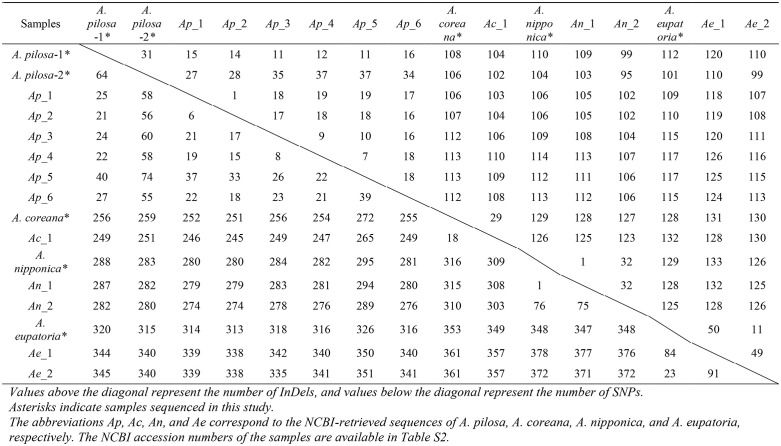

For other species, *A. coreana* accessions exhibited moderate variation with 18 SNPs and 29 InDels. *A. nipponica* showed low intraspecific diversity. For example, the sample we sequenced differed by only one SNP and one InDel from another *A. nipponica* sample (*Ap*_1). In contrast, *A. eupatoria* exhibited much higher intraspecific diversity, with 56–84 SNPs and 11–50 InDels.

Interspecific comparisons revealed substantially higher levels of variation. Between *A. pilosa* and the other species, the number of variable sites ranged from 246 to 353 SNPs and 97–120 InDels. Between *A. coreana* and the remaining two species, 306–361 SNPs and 125–132 InDels were observed. Comparisons between *A. nipponica* and *A. eupatoria* revealed 349–378 SNPs and 124–133 InDels, highlighting clear interspecific divergence.

**Fig 2:**
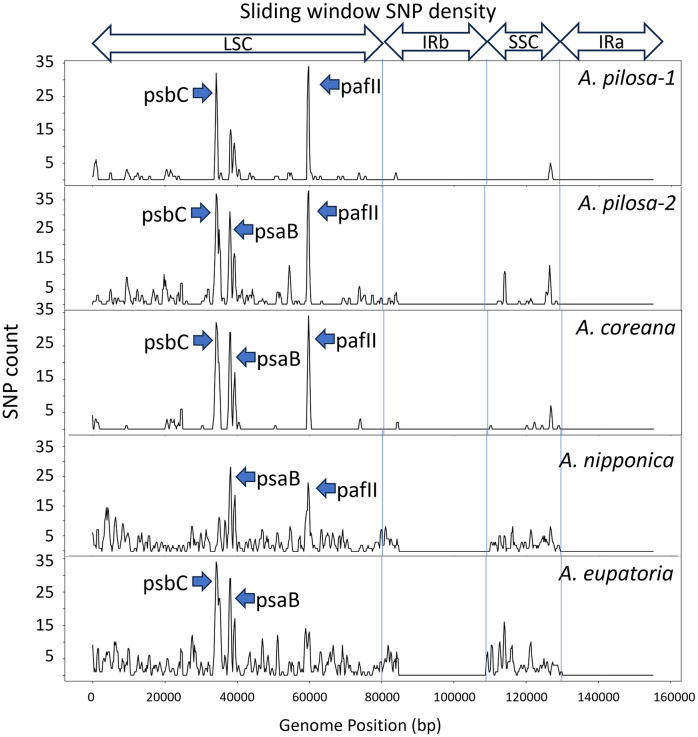
SNP density landscapes across the five *Agrimonia* plastomes. Plots illustrating SNP density, which was calculated for each sample using sliding window analysis (600 bp window and 200 bp step size). The SNP density varied across different genomic regions, revealing several localized hotspots of sequence polymorphism within the genus. Most of these highly variable regions were located in the LSC and SSC regions, whereas the IR regions remained comparatively conserved. Arrows indicate major SNP peaks located within the photosynthesis-related genes, including *psaB*, *psbC*, and *pafII*.

A detailed comparison among different species using our plastome data revealed that the positions and intensities of SNP peak varied considerably (Fig 2). For example, the SNP peak in the *psbC* region was notably less pronounced in *A. nipponica* compared to the other species. Furthermore, both *A. nipponica* and *A. eupatoria* were characterized by multiple SNP-dense segments across a broad area of the LSC region. Several SNP peaks occurred within coding regions of highly conserved photosynthesis-related genes (*psaB, psbC,* and *pafII*, the former *ycf4*), which suggest intra-individual heteroplasmy or a localized relaxation of purifying selection [24,25]. These loci may provide candidates for further investigation into the evolutionary dynamics of the *Agrimonia* plastome.

To investigate nuclear ribosomal diversity, we analyzed ten 45S nrDNA sequences, including five newly assembled in this study and five retrieved from public SRA data. Within-species comparisons revealed SNP variation of 13–28 in *A. pilosa*, eight in *A. nipponica*, and 67–71 in *A. eupatoria*. The number of InDels was minimal, ranging from none to one in *A. pilosa* and one in *A. nipponica*, while no InDels were observed in *A. eupatoria* (Table 3).

**Table 3:** Pairwise comparison of variable sites (SNPs and InDels) among *Agrimonia* 45S nrDNAs.

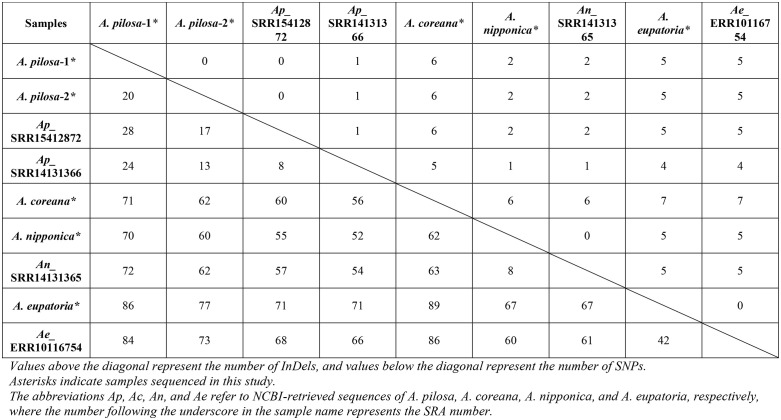

We also examined heterozygosity within nrDNA sequences among different species. The number of heterozygous sites was highest in *A. eupatoria* (n = 43), and *A. pilosa* (n = 38), followed by *A. nipponica* (n = 10) and *A. coreana* (n = 9) (S4 Table S1 File). The high heterozygosity in octoploid *A. pilosa* is likely attributable to their large number of homologous chromosomes, potentially indicating an allopolyploid origin or past hybridization events. In contrast, the lower heterozygosity detected in tetraploid *A. coreana* and *A. nipponica* may suggest an autopolyploid origin.

### Phylogenetic analysis of *Agrimonia* species

**Fig 3:**
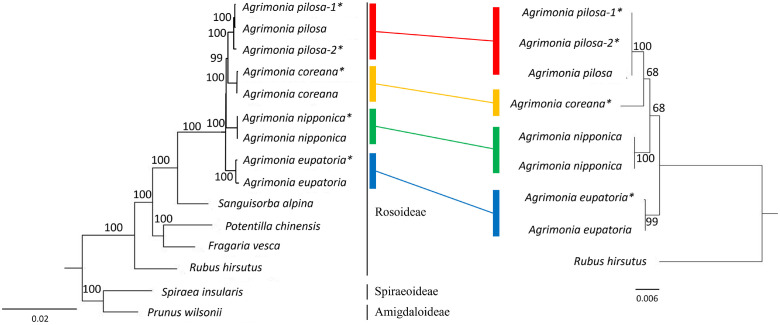
Comparison of phylogenetic trees based on plastome and 45S nrDNA sequences. Phylogenetic trees constructed from whole plastome sequences (left) and 45S nrDNA sequences (right). The plastome-based tree includes a broad selection of Rosaceae species to place *Agrimonia* in a wider phylogenetic context, with *Spiraea* and *Prunus* serving as outgroups. The nrDNA-based tree focuses on resolving relationships within the genus *Agrimonia*, using *Rubus hirsutus* as the outgroup. Both trees were generated using the neighbor-joining method with 1,000 bootstrap replicates. Bootstrap values greater than 50 are shown next to the nodes. Colored lines connect the corresponding *Agrimonia* clades, indicated by colored rectangles, in each tree.

To assess the genetic differentiation among *Agrimonia* species, we conducted a phylogenetic analysis using complete plastome sequences. This dataset included five generated in this study and four retrieved from public databases (Fig 3). To robustly test the monophyly of the genus *Agrimonia*, the analysis included other genera from Rosaceae, such as *Rubus*, *Fragaria*, *Potentilla*, and *Sanguisorba*, and *Spiraea* and *Prunus* were included as outgroups [26].

The resulting plastome-based phylogenetic tree successfully discriminated the four focal *Agrimonia* species, each forming a distinct and strongly supported group (bootstrap support ≥ 99) (Fig 3). Within the *Agrimonia* clade, *A. eupatoria* and *A. nipponica* each formed distinct and well-supported clades, while *A. pilosa* and *A. coreana* clustered together in a separate subclade. This topology suggests that *A. pilosa* and *A. coreana* are more closely related to each other than to *A. eupatoria* or *A. nipponica*, reflecting a nested pattern of divergence among the four species. These relationships are consistent with morphological similarities and prior phylogenetic studies [4]. Although the four *Agrimonia* species distributed in Korea are often misidentified due to their highly similar vegetative characteristics [4,5], the plastome sequences successfully resolved them as separate genetic lineages (Fig 3), supporting their taxonomic distinction despite morphological ambiguity.

To cross-validate these species groupings, a complementary tree was constructed using 45S nrDNA sequences from the eight *Agrimonia* accessions (Fig 3). The nrDNA tree showed a topology consistent with the plastome tree, resolving the same species clusters with strong support (bootstrap values > 68). Both the plastome- and nrDNA-based phylogenies recovered the four *Agrimonia* species as distinct and well-supported clades (Fig 3). These congruent results from two independent genomic regions indicate that the observed species boundaries are molecularly robust, at least within the Korean accessions analyzed in this study. However, considering the geographically limited sampling, broader sampling across the native range of the genus is needed to fully assess intraspecific variation and potential cryptic diversity.

### Molecular marker design and validation

To develop species-specific molecular markers for distinguishing four *Agrimonia* species commonly used and distributed in Korea, we identified polymorphisms from an analysis of 16 complete *Agrimonia* plastome sequences, comprising eight *A. pilosa*, two *A. coreana*, three *A. nipponica*, and three *A. eupatoria.* These included five newly generated plastomes in this study and 11 publicly available plastomes retrieved from NCBI. A total of eight informative loci were selected for marker design. These comprised four InDels in intergenic spacer (IGS) regions (*ndhF-rpl32*, *rps8-rpl14, psaJ-rpl20,* and *rbcL-accD*) and four SNPs in two coding genes (*rps15* and *ndhA*) and two IGS regions (*matK-rps16* and *atpB-rbcL*). Based on these variations, we designed six InDel and CAPs markers (AP1, AC1, AC2, AN1, AE1, and AE2) and two SNP markers (AP2 and AN2) (Table 4).

**Table 4:** Primer sequences and characteristics of the markers developed for differentiating *Agrimonia* species.

Marker name	Target genes	Marker type	Classification		Product Size (bp)	Primer Sequence	
AP1	*rps15*	Codominant (CAPs)	A:	*A. pilosa*	282, 168	F:	TCCCGAATATTCAACCGATTA
			B:	*A. coreana, A. nipponica, A. eupatoria*	450	R:	CCCTTTGTGTATACCTTTTCAAAT
AP2	*matK-rps16*	Dominant (SNP)	A:	*A. pilosa*	201	F:	GTTTGTTGATTAAGGCGAAGCAA
			B:	*A. coreana, A. nipponica, A. eupatoria*	–	R:	CGTTGCAATTGATGTTCGATCC
AC1	*ndhA*	Codominant (CAPs)	A:	*A. coreana*	196, 54	F:	AACCCATCGTTTTTACTTACGAA
			B:	*A. pilosa, A. nipponica, A. eupatoria*	250	R:	TGCTCCTATCCACCGATTAAA
AC2	*ndhF-rpl32*	Codominant (InDel)	A:	*A. coreana*	163	F:	GAGCAAGGATAAATAATGACAGAAC
			B:	*A. pilosa, A. nipponica, A. eupatoria*	143	R:	TGATTGGGAATAAACCGAACAAA
AN1	*rps8-rpl14*	Codominant (InDel)	A:	*A. nipponica*	210	F:	CCCTCCGAAAGAATGTTGAA
			B:	*A. pilosa, A. coreana, A. eupatoria*	182	R:	TTTTGCTTCGGAAAAGGTTC
AN2	*atpB-rbcL*	Dominant (SNP)	A:	*A. nipponica*	260	F:	GTGCAAGTCTTTTATTTGCCTTAG
			B:	*A. pilosa, A. coreana, A. eupatoria*	–	R:	GGCAGAATTCGTCCATAAACAT
AE1	*psaJ-rpl20*	Codominant (InDel)	A:	*A. eupatoria*	180	F:	CGGCACTTAAAAATCCGAAAT
			B:	*A. pilosa, A. coreana, A. nipponica*	124	R:	TGAAAGAAAGATTTCGTTGCAATA
AE2	*rbcL-accD*	Codominant (InDel)	A:	*A. eupatoria*	188	F:	TGGATCCACAATTAATCCTACG
			B:	*A. pilosa, A. coreana, A. nipponica*	148	R:	GATCCAAAAATGCAGGATCG

AP, AC, AN, and AE indicate species-specific markers designed to discriminate *A. pilosa*, *A. coreana*, *A. nipponica*, and *A. eupatoria*, respectively.

**Fig 4:**
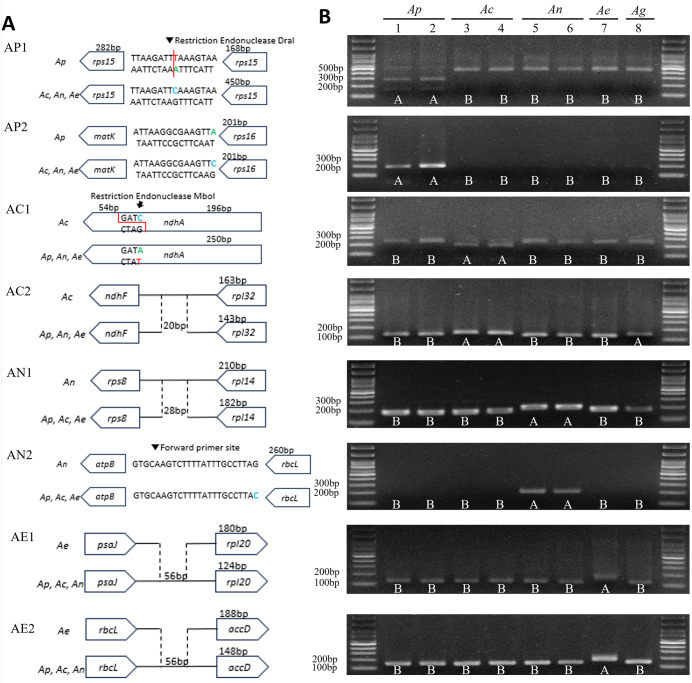
Validation of molecular markers for *Agrimonia* species identification. **(A)** Schematic representation of the eight diagnostic markers (AP1–AE2), showing their genomic locations and mutation types. Coding regions are indicated by solid boxes and intergenic regions by dashed lines. The diagrams illustrate either species-specific InDels or between *A. pilosa*, *A. coreana*, *A. nipponica*, *A. eupatoria*, and *A. gorovoii*. **(B)** Agarose gel electrophoresis of PCR products amplified from five *Agrimonia* species using the AP1–AC2 markers. The markers generated distinct, species-specific banding patterns (labeled A or **B)**, demonstrating their diagnostic utility. The species tested were *A. pilosa* (*Ap*), *A. coreana* (*Ac*), *A. nipponica* (*An*), *A. eupatoria* (*Ae*), and *A. gorovoii* (*Ag*).

To assess marker performance, the developed markers were evaluated using 36 *Agrimonia* accessions, including five individuals used for plastome sequencing (Fig 4 and S2 Fig in S2 File). The markers primarily discriminated among the four target *Agrimonia* species (*A. pilosa*, *A. coreana*, *A. nipponica*, and *A. eupatoria*), yielding species-specific plastome haplotype profiles across the validation panel. Most accessions were consistently assigned to one of these four species based on multilocus marker patterns. An additional congeneric accession, *A. gorovoii*, which was not included in the plastome assembly step, exhibited a distinct marker profile across most loci, with partial allele sharing at one locus (AC2) with *A. coreana*. This result is presented here as a preliminary observation of plastome haplotype differentiation rather than as a definitive species-level classification. Although only a single *A. eupatoria* accession was available for validation, it consistently displayed a plastome haplotype distinct from the Korean native species.

The marker set developed in this study provides a practical and reliable system for accurate identification of *Agrimonia* species commonly distributed and used in Korea, particularly for taxa that are difficult to distinguish based on vegetative morphology alone. These markers can be effectively used for molecular authentication and quality control of *Agrimonia* raw materials and may also support future germplasm management and taxonomic research within *Agrimonia*. Accordingly, the conclusions of this study are primarily applicable to *Agrimonia* species distributed and traded in Korea, while broader genus-wide application will require additional plastome-based validation.

## Supporting information

S1 File S1 Table. Sequencing and assembly information of five *Agrimonia* samples. S2 Table. Downloaded sequences from NCBI. S3 Table. Information of 31 *Agrimonia* samples used for markers validation. S4 Table. Variables and corresponding read mapping depths in 45S nrDNA sequences across the ten *Agrimonia* samples.
(XLSX)

S2 File S1 Fig. Field and specimen photographs of four *Agrimonia* species distributed in Korea. (A) *Agrimonia pilosa* Ledeb. (photographed by Jong-Soo Kang). (B) *Agrimonia coreana* Nakai (photographed by Kyung-Ah Kim). (C-D) *Agrimonia nipponica* Koidz. (photographed by Hyosun Leem). (E) *Agrimonia gorovoii* Rumjantsev (voucher specimen used for marker validation; specimen deposited in the Korean Herbarium of Standard Herbal Resources; herbarium code KIOM). S2 Fig. Validation of PCR-based markers for the identification of *Agrimonia* species. Agarose gel electrophoresis shows the species-specific amplification of diagnostic markers using genomic DNA from five *Agrimonia* species: *A. pilosa* (Ap), *A. coreana* (Ac), *A. nipponica* (An), *A. eupatoria* (Ae), and *A. gorovoii* (Ag). (A) Amplification patterns using the marker set AP1–AC2. (B) Amplification patterns using the marker set AN1–AE2.
(PDF)

S3 File Raw images. (PDF)
