# Supplementary material for: Super-barcoding of four Agrimonia species distributed in Korea based on complete plastid genomes and nuclear ribosomal DNAs
Source: PLoS One. 2026 Feb 13;21(2):e0341151. doi: 10.1371/journal.pone.0341151 (PMC12904433; doi:10.1371/journal.pone.0341151)
Supplement: S2 File — S1 Fig. Field and specimen photographs of four Agrimonia species distributed in Korea. (A) Agrimonia pilosa Ledeb. (photographed by Jong-Soo Kang). (B) Agrimonia coreana Nakai (photographed by Kyung-Ah Kim). (C-D) Agrimonia nipponica Koidz. (photographed by Hyosun Leem). (E) Agrimonia gorovoii Rumjantsev (voucher specimen used for marker validation; specimen deposited in the Korean Herbarium of Standard Herbal Resources; herbarium code KIOM). S2 Fig. Validation of PCR-based markers for the identification of Agrimonia species. Agarose gel electrophoresis shows the species-specific amplification of diagnostic markers using genomic DNA from five Agrimonia species: A. pilosa (Ap), A. coreana (Ac), A. nipponica (An), A. eupatoria (Ae), and A. gorovoii (Ag). (A) Amplification patterns using the marker set AP1–AC2. (B) Amplification patterns using the marker set AN1–AE2. (PDF) [file pone.0341151.s002.pdf]

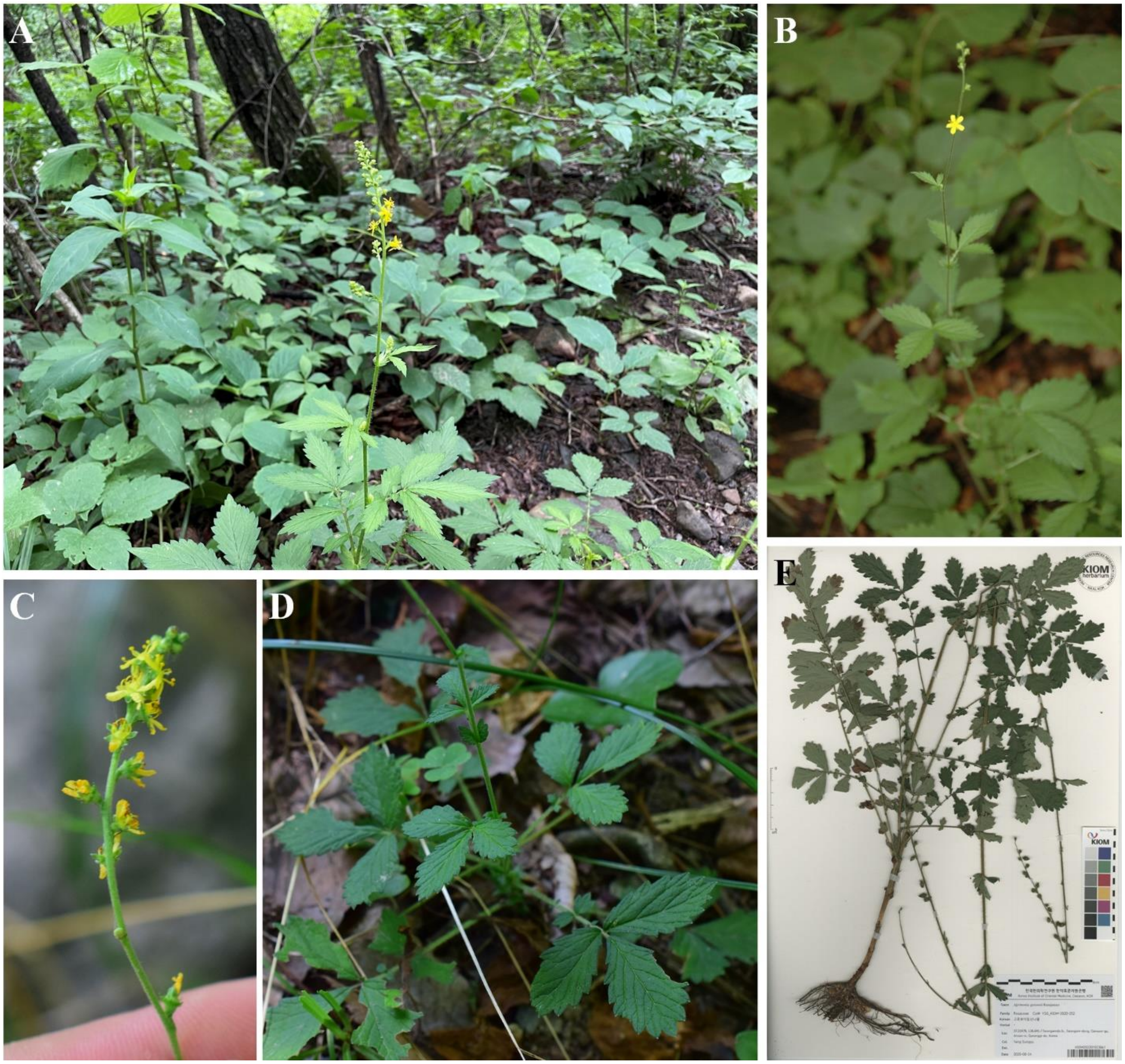

S1 Fig. Field and specimen photographs of four *Agrimonia* species distributed in Korea. (A) *Agrimonia pilosa* Ledeb. (photographed by Jong-Soo Kang). (B) *Agrimonia coreana* Nakai (photographed by Kyung-Ah Kim). (C-D) *Agrimonia nipponica* Koidz. (photographed by Hyosun Leem). (E) *Agrimonia gorovoii* Rumjantsev (voucher specimen used for marker validation; specimen deposited in the Korean Herbarium of Standard Herbal Resources; herbarium code KIOM).

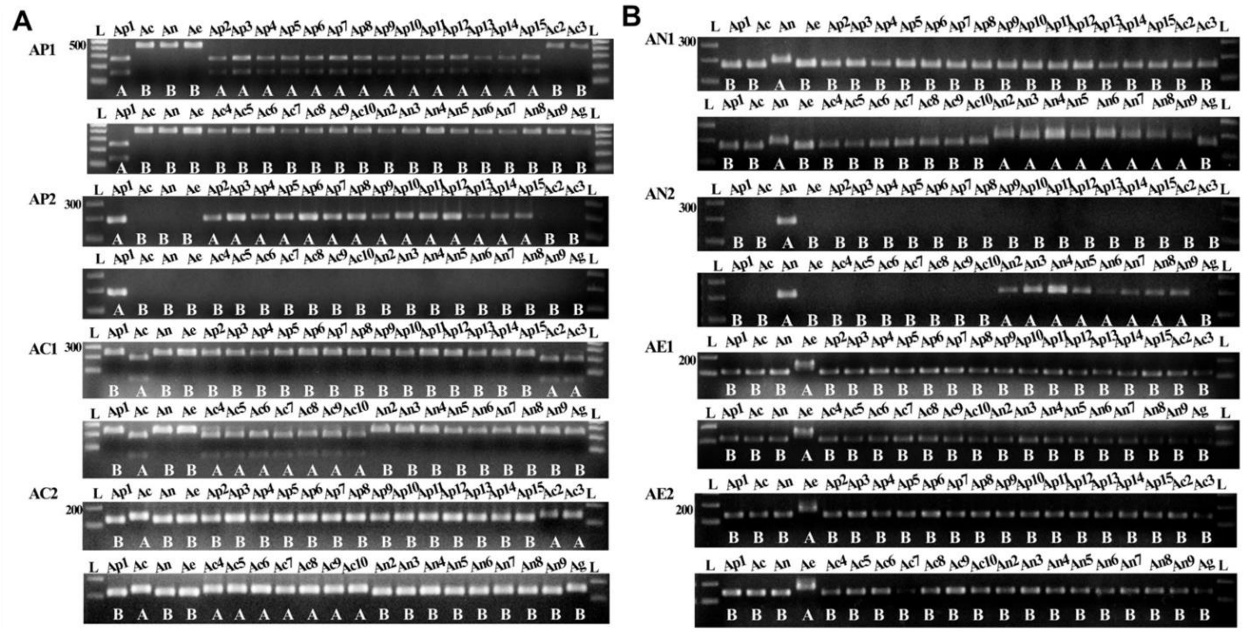

S2 Fig. Validation of PCR-based markers for the identification of *Agrimonia* species. Agarose gel electrophoresis shows the species-specific amplification of diagnostic markers using genomic DNA from five *Agrimonia* species: *A. pilosa* (Ap), *A. coreana* (Ac), *A. nipponica* (An), *A. eupatoria* (Ae), and *A. gorovoii* (Ag). (A) Amplification patterns using the marker set AP1–AC2. (B) Amplification patterns using the marker set AN1–AE2.
