# Supplementary material for: Super-barcoding of four Agrimonia species distributed in Korea based on complete plastid genomes and nuclear ribosomal DNAs
Source: PLoS One. 2026 Feb 13;21(2):e0341151. doi: 10.1371/journal.pone.0341151 (PMC12904433; doi:10.1371/journal.pone.0341151)
Supplement: S3 File — (PDF) [file pone.0341151.s003.pdf]

Original uncropped gel images used in Fig 3

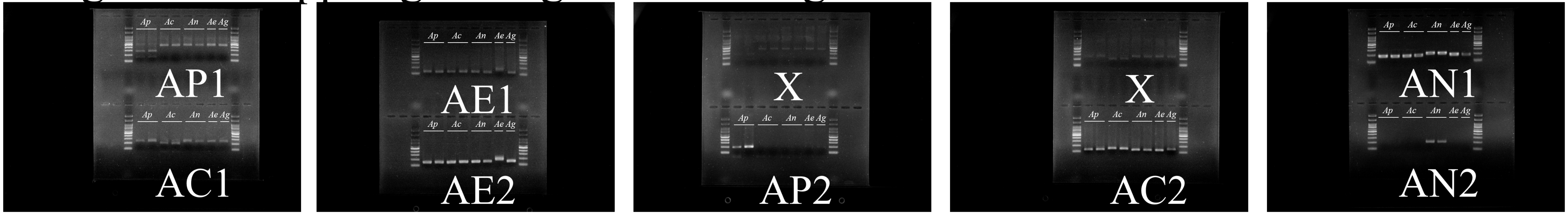

Original uncropped gel images used in S1 Fig

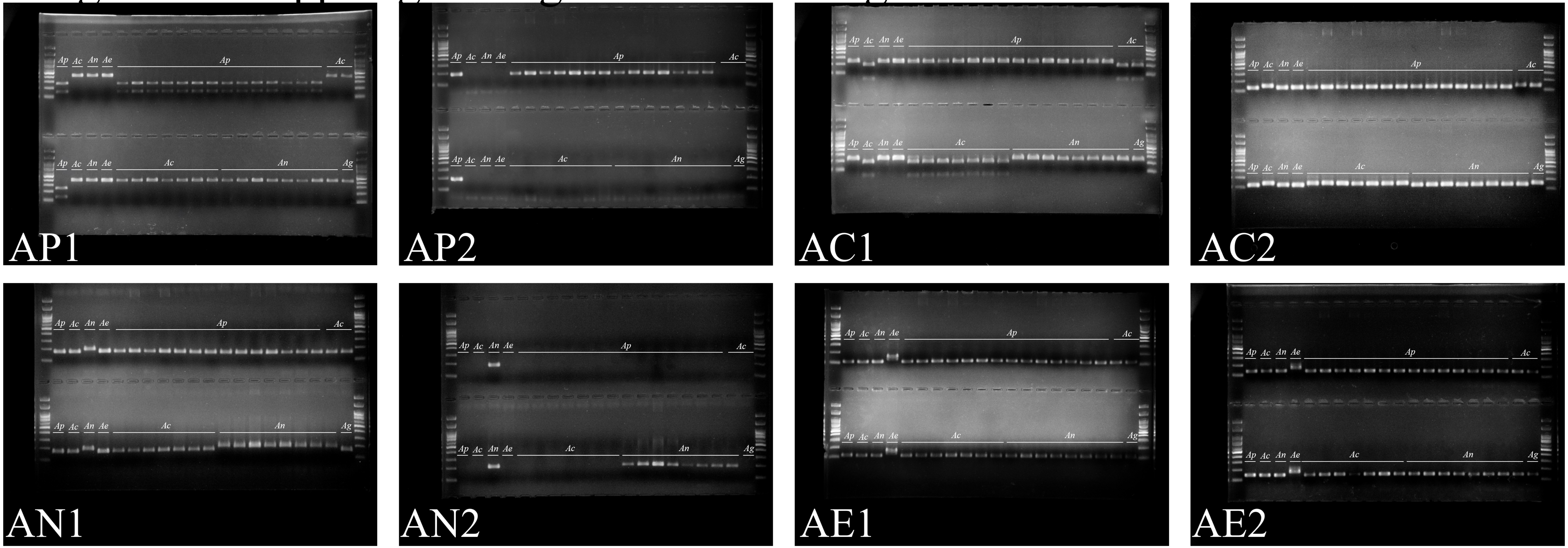

Ap : Agrimonia pilosa, Ac: Agrimonia coreana, An: Agrimonia nipponica, Ae: Agrimonia eupatoria, Ag: Agrimonia gorovoii
